# Hypertrophic Cardiomyopathy With Left Ventricular Systolic Dysfunction: Insights From the SHaRe Registry

**DOI:** 10.1161/CIRCULATIONAHA.119.044366

**Published:** 2020-03-31

**Authors:** Peter Marstrand, Larry Han, Sharlene M. Day, Iacopo Olivotto, Euan A. Ashley, Michelle Michels, Alexandre C. Pereira, Samuel G. Wittekind, Adam Helms, Sara Saberi, Daniel Jacoby, James S. Ware, Steven D. Colan, Christopher Semsarian, Jodie Ingles, Neal K. Lakdawala, Carolyn Y. Ho

**Affiliations:** 1 Cardiovascular Division, Brigham and Women’s Hospital, Boston, MA (P.M., N.K.L., C.Y.H.).; 2 Department of Biostatistics, Harvard T.H. Chan School of Public Health, Boston, MA (L.H.).; 3 Department of Medicine, University of Pennsylvania, Philadelphia (S.M.D.).; 4 Cardiomyopathy Unit, Careggi University Hospital, Florence, Italy (I.O.).; 5 Stanford Center for Inherited Heart Disease, CA (E.A.A.).; 6 Department of Cardiology, Thoraxcenter, Erasmus Medical Center Rotterdam, the Netherlands (M.M.).; 7 Heart Institute (InCor), University of São Paulo Medical School, Brazil (A.C.P.).; 8 Cincinnati Children’s Hospital Medical Center, Heart Institute, OH (S.G.W.).; 9 Department of Internal Medicine, University of Michigan, Ann Arbor (A.H., S.S.).; 10 Yale University, New Haven, CT (D.J.).; 11 National Heart and Lung Institute and Royal Brompton Cardiovascular Research Centre, Imperial College London, United Kingdom (J.S.W.).; 12 Department of Cardiology, Boston Children’s Hospital, MA (S.D.C.).; 13 Agnes Ginges Centre for Molecular Cardiology at Centenary Institute, University of Sydney, Australia (C.S., J.I.).; 14 Department of Cardiology, Herlev-Gentofte Hospital, University Hospital of Copenhagen, Denmark (P.M.).

**Keywords:** cardiomyopathy, hypertrophic, genetics, heart failure, prognosis, ventricular dysfunction

## Abstract

The term “end stage” has been used to describe hypertrophic cardiomyopathy (HCM) with left ventricular systolic dysfunction (LVSD), defined as occurring when left ventricular ejection fraction is <50%. The prognosis of HCM-LVSD has reportedly been poor, but because of its relative rarity, the natural history remains incompletely characterized.

**Methods:**

Data from 11 high-volume HCM specialty centers making up the international SHaRe Registry (Sarcomeric Human Cardiomyopathy Registry) were used to describe the natural history of patients with HCM-LVSD. Cox proportional hazards models were used to identify predictors of prognosis and incident development.

**Results:**

From a cohort of 6793 patients with HCM, 553 (8%) met the criteria for HCM-LVSD. Overall, 75% of patients with HCM-LVSD experienced clinically relevant events, and 35% met the composite outcome (all-cause death [n=128], cardiac transplantation [n=55], or left ventricular assist device implantation [n=9]). After recognition of HCM-LVSD, the median time to composite outcome was 8.4 years. However, there was substantial individual variation in natural history. Significant predictors of the composite outcome included the presence of multiple pathogenic/likely pathogenic sarcomeric variants (hazard ratio [HR], 5.6 [95% CI, 2.3–13.5]), atrial fibrillation (HR, 2.6 [95% CI, 1.7–3.5]), and left ventricular ejection fraction <35% (HR, 2.0 [95% CI, 1.3–2.8]). The incidence of new HCM-LVSD was ≈7.5% over 15 years. Significant predictors of developing incident HCM-LVSD included greater left ventricular cavity size (HR, 1.1 [95% CI, 1.0–1.3] and wall thickness (HR, 1.3 [95% CI, 1.1–1.4]), left ventricular ejection fraction of 50% to 60% (HR, 1.8 [95% CI, 1.2, 2.8]–2.8 [95% CI, 1.8–4.2]) at baseline evaluation, the presence of late gadolinium enhancement on cardiac magnetic resonance imaging (HR, 2.3 [95% CI, 1.0–4.9]), and the presence of a pathogenic/likely pathogenic sarcomeric variant, particularly in thin filament genes (HR, 1.5 [95% CI, 1.0–2.1] and 2.5 [95% CI, 1.2–5.1], respectively).

**Conclusions:**

HCM-LVSD affects ≈8% of patients with HCM. Although the natural history of HCM-LVSD was variable, 75% of patients experienced adverse events, including 35% experiencing a death equivalent an estimated median time of 8.4 years after developing systolic dysfunction. In addition to clinical features, genetic substrate appears to play a role in both prognosis (multiple sarcomeric variants) and the risk for incident development of HCM-LVSD (thin filament variants).

## Abstract 2

Supplemental Digital Content is available in the text.

**Clinical Perspective**

**What Is New?**

- Left ventricular systolic dysfunction develops in ≈8% of patients with hypertrophic cardiomyopathy (HCM).
- From recognition of HCM with left ventricular systolic dysfunction, the estimated median time to death, transplantation, or need for left ventricular assist device is 8.4 years.
- Risk factors of poor prognosis for patients with HCM with left ventricular systolic dysfunction are multiple pathogenic/likely pathogenic sarcomeric variants, atrial fibrillation, and left ventricular ejection fraction <35%.
- Patients with HCM with pathogenic sarcomeric variants (particularly in thin filament genes), increased left ventricular wall thickness, left ventricular dilation, and borderline low ejection fraction (50%–59%) are at higher risk for developing HCM with left ventricular systolic dysfunction.

**What Are the Clinical Implications?**

- Assessing both genetic and clinical risk factors can improve stratification and prognostication of patients with HCM.
- Patients with left ventricular ejection fraction of 50% to 59% have an increased risk for further decline in systolic function and may benefit from closer follow-up.

Hypertrophic cardiomyopathy (HCM) is the most common inherited cardiac disease, with an estimated prevalence of 1 in 500 in the adult population.^1–3^ Pathogenic variants in genes that encode cardiac sarcomeric proteins are responsible for causing disease in roughly 60% of familial and 20% to 30% of apparently sporadic HCM.^4^ Left ventricular (LV) systolic function is characteristically normal to hyperdynamic in HCM. However, prior studies have suggested that 4% to 9% of patients develop systolic dysfunction, defined by LV ejection fraction (LVEF) <50% (herein referred to as HCM with LV systolic dysfunction [LVSD]). This complication of disease has previously been called “end-stage” or “burnt-out” HCM.^5–8^ HCM-LVSD is typically accompanied by diffuse myocardial fibrosis, although LV wall thinning and cavity enlargement may also be present.^6,9^ The reported prognosis of patients with HCM-LVSD has been quite poor, with mortality as high as 11%/year.^6^ However, because of its relative rarity, the natural history remains incompletely characterized. There is a clear need to better understand HCM-LVSD to improve risk stratification and to inform clinical management. In this study, we leverage the international SHaRe (Sarcomeric Human Cardiomyopathy Registry),^10^ analyzing data on nearly 7000 patients with HCM, to better describe the prevalence and natural history of HCM-LVSD, to identify predictors of prognosis, and to identify features that predict incident development of systolic dysfunction.

## Methods

SHaRe is an international collaborative consortium of high-volume HCM centers that maintain longitudinal databases capturing phenotypic, genetic, and clinical outcomes data on patients with HCM and their families. The structure of the registry and initial findings have previously been described in detail.^10^ Briefly, definitions for phenotypic features and clinical outcomes were harmonized, and site data are mapped to a secure, centralized data set. Historical events that occurred before the initial visits to SHaRe sites are carefully ascertained and vetted for accuracy through detailed patient history and medical record review. Prospective longitudinal data are captured by sites as they occur or during clinical encounters and uploaded quarterly from site databases. Institutional review and ethics approval were obtained in accordance with applicable site policies. This study analyzed data from 1960 through March 2019 from 11 different sites around the world (Brigham and Women’s Hospital, Boston, MA; Boston Children’s Hospital, MA; University of Michigan, Ann Arbor; Cincinnati Children’s Hospital, OH; Yale-New Haven Hospital, CT; Stanford University, CA; Cardiomyopathy Unit, University of Florence, Italy; Erasmus Medical Center, Rotterdam, the Netherlands; Royal Brompton Hospital, London, United Kingdom; University of São Paulo, Brazil; University of Sydney, Australia). The data cannot be made available to other researchers for purposes of reproducing or replicating the procedure because of constraints related to human subjects research. Analytical methods can be made available on request.

### Study Population

Patients were included in this study if they had at least 1 complete echocardiographic study at a SHaRe site and a site-designated diagnosis of HCM, defined as unexplained LV hypertrophy with a maximal LV wall thickness >15 mm or >13 mm in members of families with HCM (or equivalent LV wall thickness *z* score in pediatric patients). Extracardiac features, family history, and genotype were integrated to allow accurate and informed diagnosis by experienced clinicians. Genetic testing was performed at sites using different platforms available over time, focusing on the 8 sarcomeric genes definitively associated with HCM (*MYBPC3*, *MYH7*, *TNNT2*, *TNNI3*, *TPM1*, *MLY2*, *MYL3*, and *ACTC*). Sites used current criteria^11^ to classify variants as pathogenic or likely pathogenic (denoted SARC+; including patients with >1 pathogenic/likely pathogenic variant), a variant of unknown significance (denoted SARC VUS), or likely benign/benign (included as SARC− with patients with no clinically relevant variants identified on genetic testing, denoted SARC−). Sarcomeric variants with discordant classification among sites underwent additional systematic review by a subgroup of investigators (C.Y.H., J.S.W., S.M.D.) to adjudicate and standardize classification. Patients were excluded if they had potentially pathogenic variants in genes encoding nonsarcomeric proteins such as *GLA* or *LAMP2*, indicating the presence of metabolic or storage disease or other phenocopies of HCM. Initial clinical characteristics represent data at first evaluation at a SHaRe site. All cardiac dimensions and function were based on echocardiographic measurements.

### Outcomes

Patients were designated as having HCM-LVSD at the first documentation of an echocardiographic LVEF <50% on a clinically performed echocardiographic study. To identify predictors of prognosis in HCM-LVSD, patients were followed from recognition of HCM-LVSD until last follow-up or meeting the composite outcome of all-cause mortality, cardiac transplantation, or implantation of an LV assist device (LVAD). Development of atrial fibrillation and New York Heart Association (NYHA) functional class III/IV symptoms were also assessed. Analyses to identify factors associated with incident HCM-LVSD included genotyped patients with LVEF ≥50% and no history of septal reduction at or before their initial SHaRe visit. Patients with existing HCM-LVSD at their initial SHaRe evaluation were excluded from this model. Sensitivity analysis was performed to compare septal reduction therapies (SRTs) in patients diagnosed with HCM before and after January 2000 to limit analysis to patients who were diagnosed with HCM and underwent procedures in the current era.

### Statistical Analysis

Normally distributed data were expressed as mean±SD and compared with the Student *t* test. Nonnormally distributed data were expressed as median and interquartile range and compared with the Wilcoxon rank-sum test. Differences in categorical variables were calculated with the χ^2^ test. The Kaplan-Meier method with log-rank test for significance was used to estimate the cumulative incidence and time to event of the end points of interest, starting from the time of the initial visit to a SHaRe site. To adjust for baseline characteristics, Cox proportional hazards models were developed, requiring a minimum of 10 events (incident HCM-LVSD or composite outcome) per covariate included.^12^ Results of Cox regressions were reported as adjusted hazard ratios (HRs) with 95% CIs. Analyses were depicted in forest plots, and values of *P*<0.05 were considered statistically significant. If patients were missing echocardiographic measures at the first SHaRe evaluation, data were imputed from subsequent echocardiographic studies if the echocardiogram was performed before the development of HCM-LVSD. Patients missing key echocardiographic data in all of their studies in SHaRe were omitted from Cox regression models. R version 3.5.2 was used for all analyses.

## Results

### Prevalence and Clinical Features of HCM-LVSD

Of 7594 patients with HCM receiving care at a SHaRe site between 1960 and March 2019, 6793 met inclusion criteria and were analyzed in this study. HCM-LVSD was present in 553 patients (8.1% of the cohort; Table 1), including 203 who already had LVEF <50% at initial SHaRe evaluation (prevalent HCM-LVSD) and 350 who developed HCM-LVSD during follow-up (incident HCM-LVSD). Men represented 62.2% of the overall cohort, and there was no significant difference in the proportion of male and female patients with and without LVSD. Collectively, patients with HCM-LVSD (incident and prevalent cases combined) were ≈6.5 years younger at diagnosis than patients with HCM without systolic dysfunction (35.6 years versus 42.1 years; *P*<0.001) with no significant difference between the prevalent and incident HCM-LVSD groups. It is notable that follow-up was longer in patients with incident HCM-LVSD compared with patients without systolic dysfunction (median, 9.8 years versus 2.9 years; *P*<0.001) and patients with prevalent HCM-LVSD (median, 3.2 years; *P*<0.001). In a comparison of genotyped patients (n=4224), patients with HCM-LVSD were more likely to have sarcomeric disease than patients without systolic dysfunction (241 of 394 [61.2%] versus 1767 of 3830 [46.1%]; *P*<0.001).

**Table 1.**
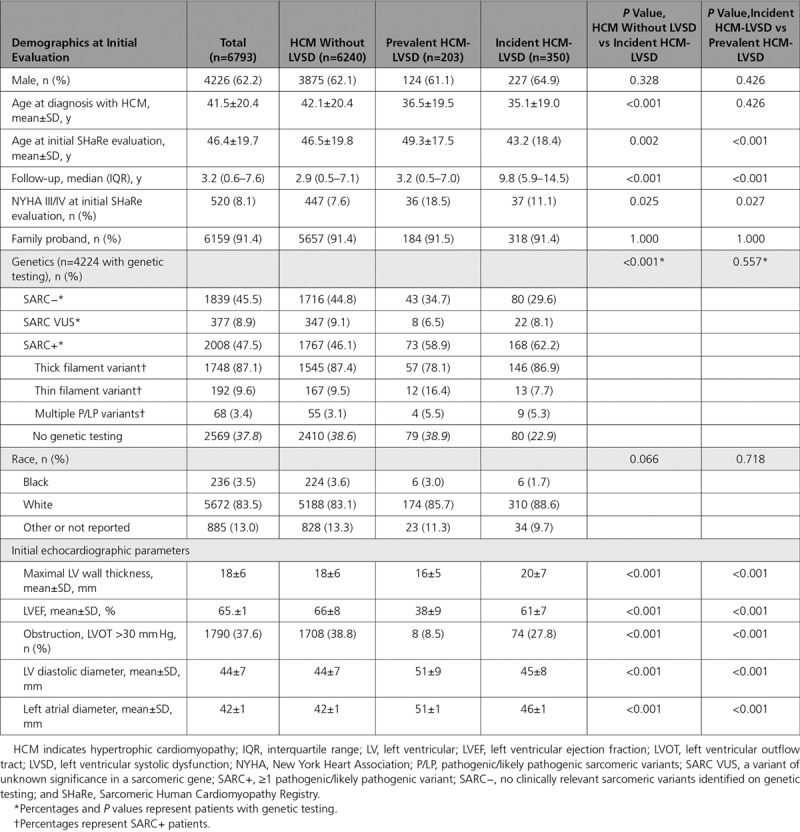
Clinical Characteristics of Patients at the Initial SHaRe Evaluation

As Table 1 shows, patients with incident HCM-LVSD had greater maximal LV wall thickness (20±7 mm versus 18±6 mm; *P*<0.001), LV diastolic diameter (45±8 mm versus 44±7 mm; *P*<0.001), and left atrial diameter (46±1 mm versus 42±1 mm; *P*<0.001) at initial SHaRe evaluation compared with patients with HCM who did not develop systolic dysfunction. Although patients with incident HCM-LVSD did not have systolic dysfunction at their initial visit, LVEF was significantly lower than in patients with HCM who did not develop systolic dysfunction (61±7% versus 66±8%; *P*<0.001). Patients with incident HCM-LVSD were also more likely to have NYHA class III/IV symptoms at the initial SHaRe visit (11.1% versus 7.6%; *P*=0.025) but less likely to have obstructive physiology (defined as LV outflow tract >30 mm Hg; 27.8% versus 38.8%; *P*<0.001).

Raw counts and HRs of all events experienced by patients with HCM-LVSD and HCM without LVSD are presented in Table 2. HRs are adjusted for age, sex, and follow-up time from the initial SHaRe evaluation. Patients with HCM-LVSD had a markedly higher prevalence of cardiac transplantation (11.4% versus 0.7%) and LVAD implantation (1.6% versus 0.1%; *P*<0.001 for all comparisons). Patients with HCM-LVSD were more likely to experience all-cause death (25.0% versus 6.7%), implantable cardioverter-defibrillator (ICD) implantation (54.4% versus 21.6%), and appropriate ICD firing (25.2% versus 12.1%; *P*<0.001). Atrial fibrillation (49.3% versus 20.9%) and stroke (8.4% versus 2.3%) were also more common in patients with HCM-LVSD.

**Table 2.**
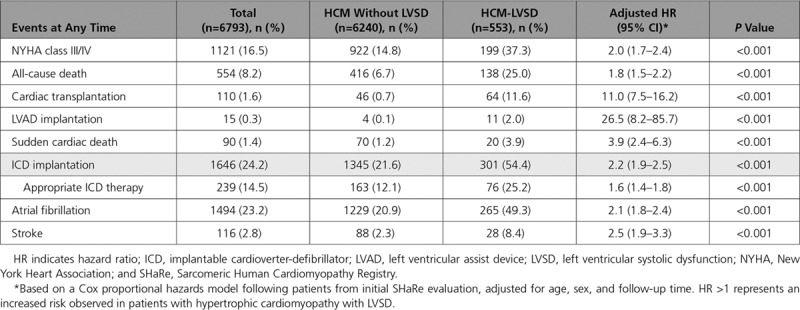
Comparison of Events in Patients With and Without LVSD

A greater proportion of patients with HCM-LVSD underwent SRT compared with patients without LVSD (27.3% versus 17.5%; *P*<0.001; Table 3). The majority of procedures were myectomy (78% of SRT procedures). The proportion undergoing alcohol septal ablation did not differ between groups (4.0% versus 3.2%; *P*=0.34), but patients with HCM-LVSD were more likely to have had both myectomy and alcohol ablation performed (1.6% versus 0.6%; *P*<0.001).

**Table 3.**
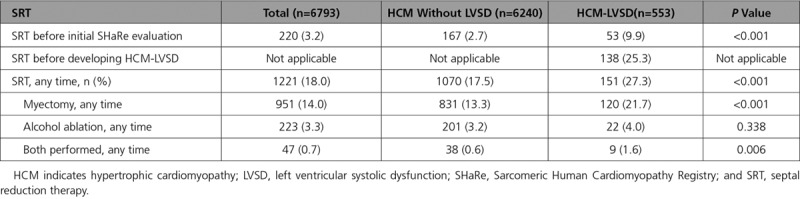
Comparison of Patients With and Without LVSD Who Underwent Invasive SRT

### Natural History of HCM-LVSD

We characterized natural history by focusing on our cohort of 553 patients with HCM-LVSD. As Table 4 shows, patients with HCM-LVSD were a mean age of 35.6±19.2 years when diagnosed with HCM and 50.3±17.9 years when recognized to have LVEF <50%. The mean LVEF at presentation with HCM-LVSD was 40±8%, and 27.1% of patients had an LVEF <35% at presentation. Thirty percent (165 of 553) had NYHA class III/IV symptoms at presentation with HCM-LVSD.

**Figure 1.**
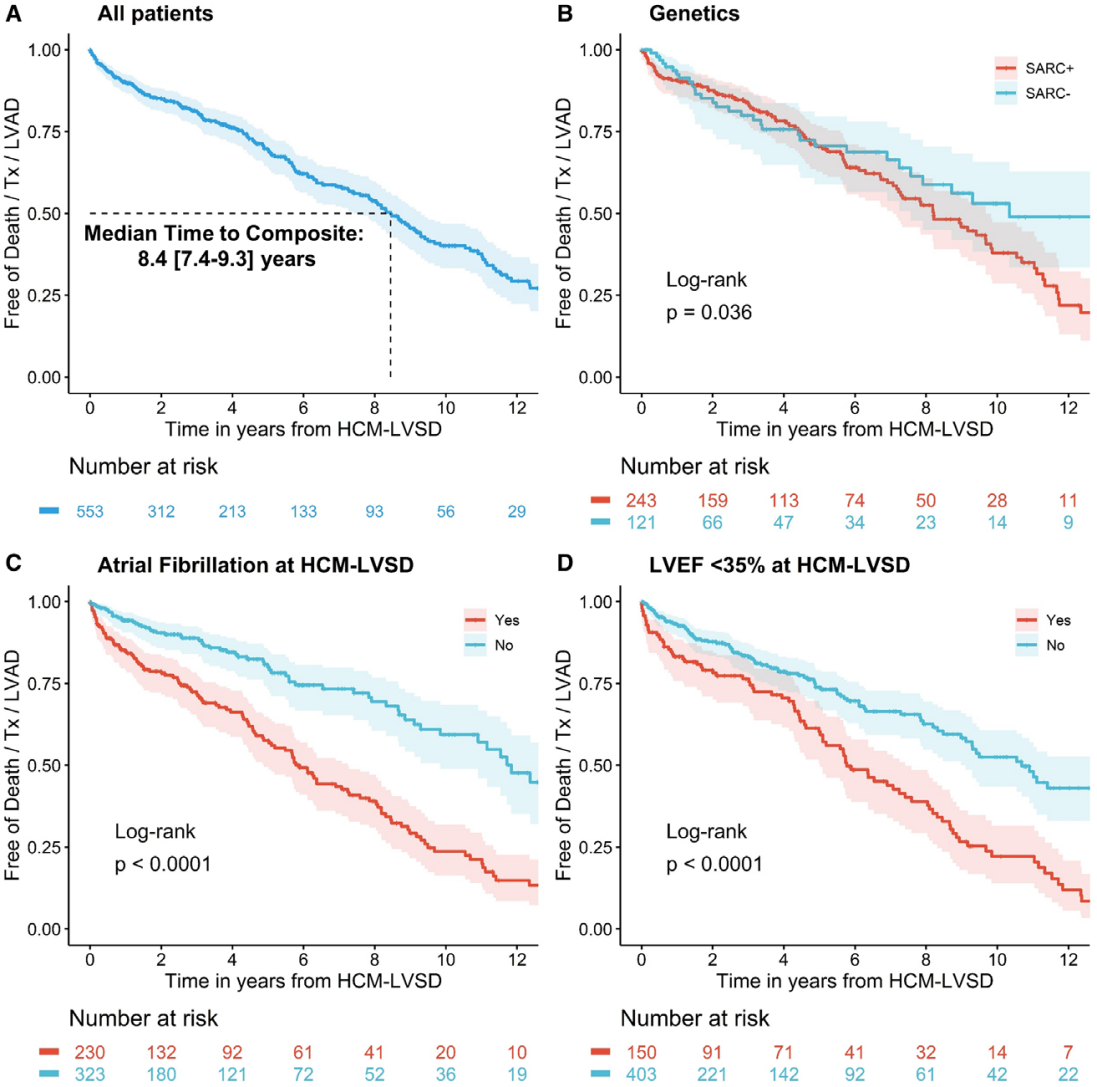
**Kaplan-Meier curves for reaching the composite outcome from the time of diagnosis of hypertrophic cardiomyopathy with left ventricular systolic dysfunction (HCM-LVSD).** **A**, The estimated median time to occurrence of the composite outcome is 8.4 years (95% CI, 7.4–9.3 years). Patients with (**B**) pathogenic sarcomeric variants (SARC+), (**C**) atrial fibrillation, or (**D**) left ventricular ejection fraction (LVEF) <35% at HCM-LVSD diagnosis all have worse outcomes. LVAD indicates left ventricular assist device; SARC−, no clinically relevant sarcomeric variants; and Tx, cardiac transplantation.

Table 5 lists clinical events associated with the total HCM-LVSD cohort, both before developing systolic dysfunction and during follow-up. Overall, 74.7% of patients with HCM-LVSD experienced clinically relevant events. At the time of HCM-LVSD presentation, 41.6% of patients had atrial fibrillation and 39.1% had an ICD. During follow-up, 192 patients (34.7%) with HCM-LVSD met the composite outcome (all-cause death [n=128], cardiac transplantation [n=55], or LVAD implantation [n=9]). In addition, 25% of those with an ICD received an appropriate therapy (40 of 186 [22%] patients with LVEF <35% and 36 of 397 [9%] patients with LVEF >35%; *P*<0.001). The estimated median time from recognition of HCM-LVSD to composite outcome was 8.4 years (95% CI, 7.4–9.3; Figure 1A). Assessing death alone as an outcome showed that 138 patients (25%) with HCM-LVSD died, including 10 patients who died after cardiac transplantation or LVAD implantation. The median time to death was 11.4 years (95% CI, 9.3–14.9 years) after the development of systolic dysfunction. Patients with HCM-LVSD at the initial evaluation had a prognosis from the time of HCM-LVSD similar to those who developed incident HCM-LVSD during follow-up (data not shown).

**Table 4.**
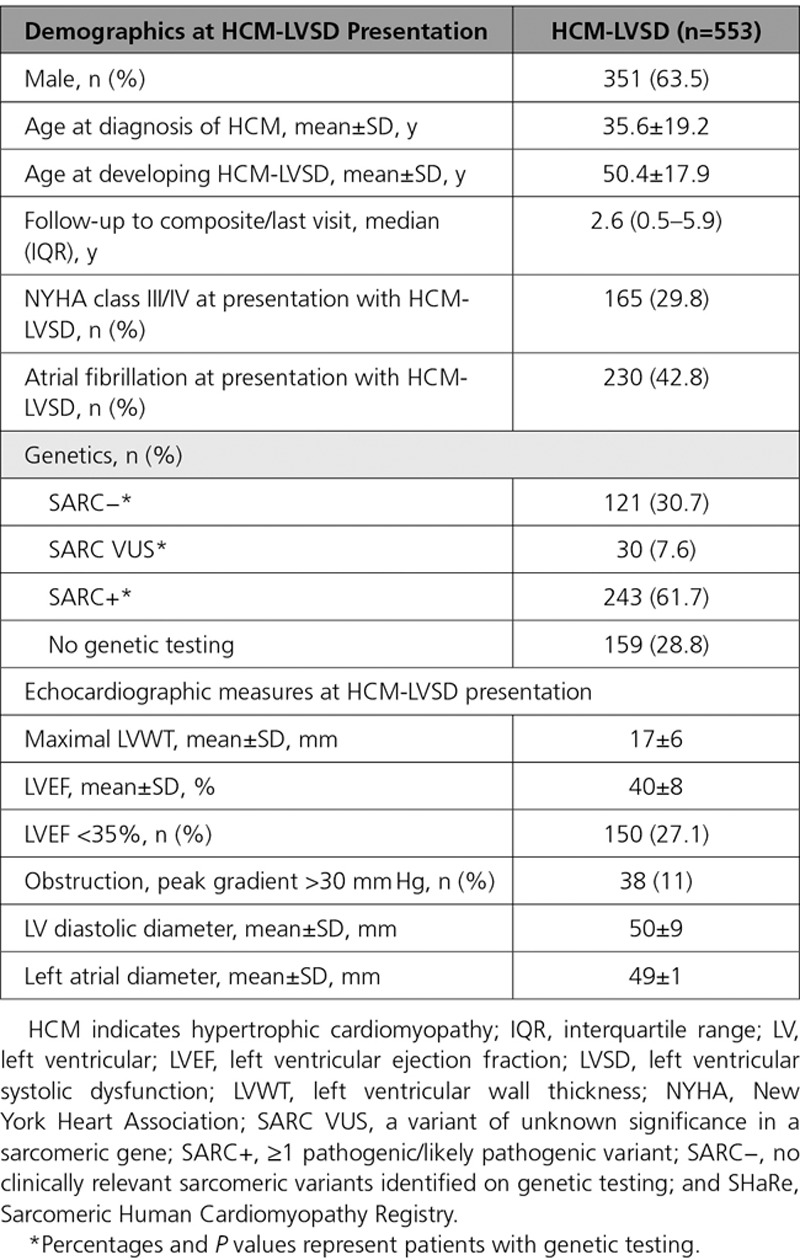
Clinical Characteristics at Time of Presentation With HCM-LVSD

**Table 5.**
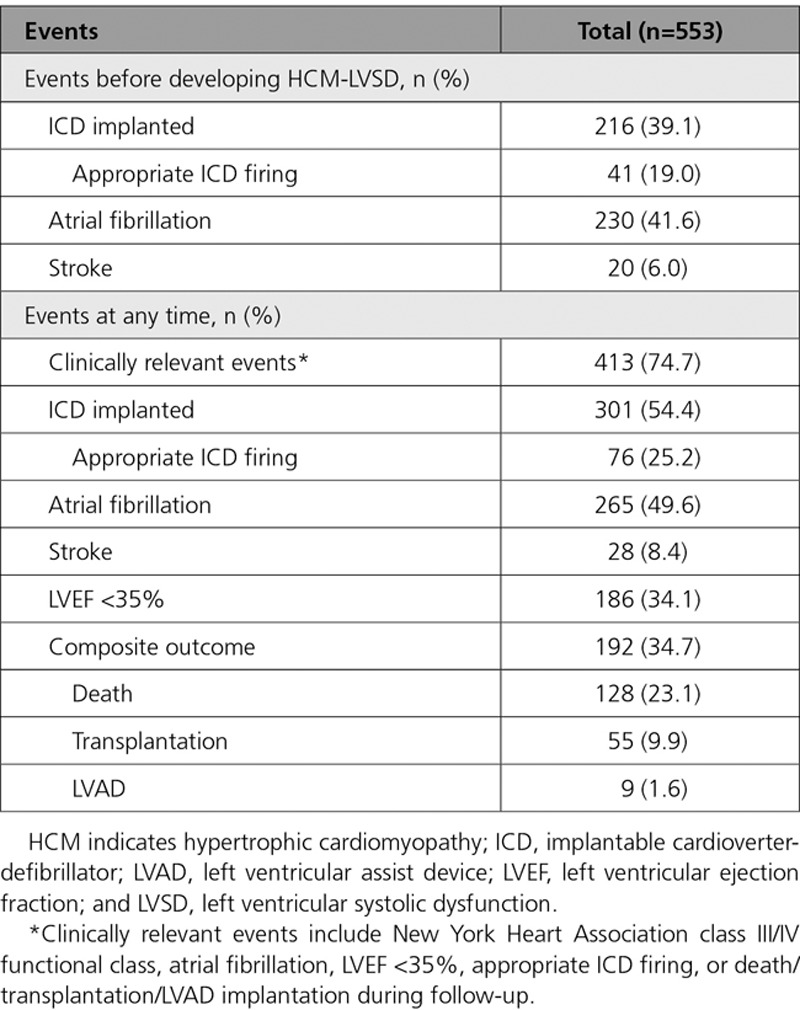
Overview of Adverse Events at Time of HCM-LVSD and at Any Time in Patients With HCM-LVSD

**Figure 2.**
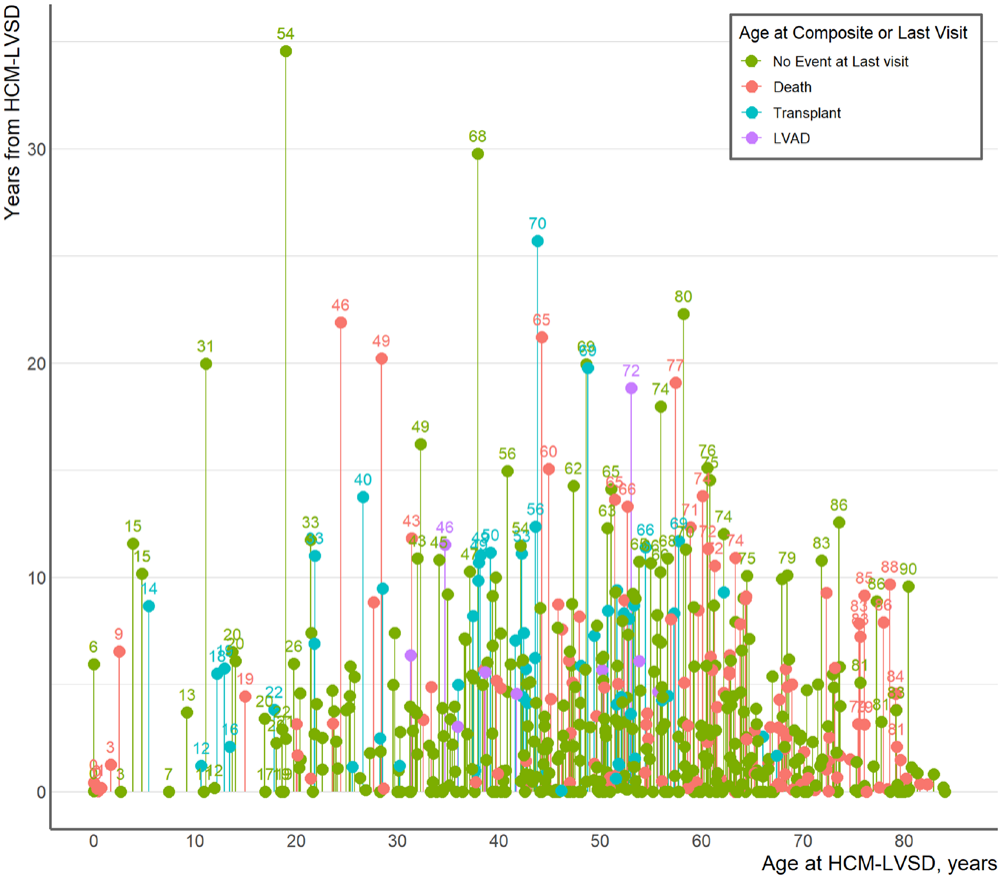
**The natural history of hypertrophic cardiomyopathy with left ventricular systolic dysfunction (HCM-LVSD) is variable.** Individual-level events are shown in 553 patients with HCM-LVSD from the time of presentation with HCM-LVSD to the time of event or last visit. The *x* axis represents the patient’s age at diagnosis of HCM-LVSD; the *y* axis represents years until death (red), cardiac transplantation (blue), left ventricular assist device (LVAD) implantation (purple), or last visit (green). Age at event or last visit is shown above mark for a limited number of patients.

Clinical course varied substantially from patient to patient. As Figure 2 illustrates, many patients with HCM-LVSD across a broad spectrum of ages do not experience the composite outcome, whereas many others at similar ages and duration of HCM-LVSD experienced serious outcomes.

### Predictors of Prognosis in HCM-LVSD

**Figure 3.**
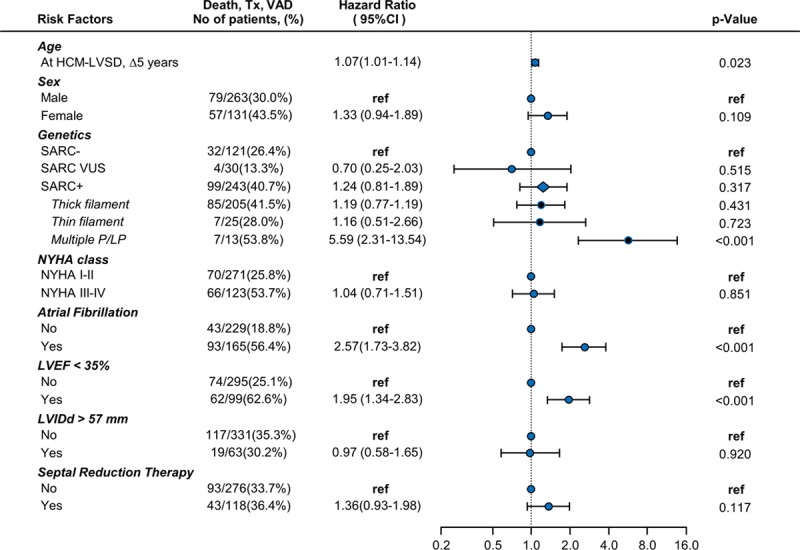
**Forest plot depicting risk predictors for developing the composite outcome of all-cause mortality, cardiac transplantation (Tx), or left ventricular assist device (VAD) implantation in 394 genotyped patients with hypertrophic cardiomyopathy with left ventricular systolic dysfunction (HCM-LVSD) experiencing 136 composite events.** Unadjusted number of events per variable is shown in the second column. Hazard ratios are adjusted for all included risk factors. The diamond symbol in the cohort with ≥1 pathogenic/likely pathogenic variant (SARC+) represents the composite effect of thin filament, thick filament, and multiple pathogenic/likely pathogenic (P/LP) sarcomere variants. LVEF indicates left ventricular ejection fraction; LVIDd, left ventricular diastolic diameter; NYHA, New York Heart Association; ref, referent; SARC−, no clinically relevant sarcomere variants identified on genetic testing; and SARC VUS, a variant of unknown significance in a sarcomere gene.

Among the 553 patients with HCM-LVSD, carrying a pathogenic sarcomeric variant (*P*=0.039), concomitant atrial fibrillation (*P*<0.001), and severe LV dysfunction at recognition of HCM-LVSD (LVEF <35%, *P*<0.001) were each associated with a higher likelihood and earlier occurrence of the composite outcome in univariate analyses (Figure 1B-D). To identify independent predictors of poor prognosis, we performed a multivariate analysis for 394 of the 553 patients with HCM-LVSD who were genotyped (Figure 3). Of this genotyped cohort, 136 (34.5%) experienced the composite outcome. Atrial fibrillation (HR 2.6 [95% CI, 1.8–3.8]) and LVEF <35% at recognition of HCM-LVSD (HR 2.0 [95% CI, 1.4–2.8]) remained independent predictors in this model. A single sarcomere gene mutation was not an independent predictor, suggesting that mutation status covaries with atrial fibrillation or LVEF <35%. However, patients with multiple pathogenic/likely pathogenic sarcomeric variants had the highest risk for the composite outcome (HR, 5.6 [95% CI, 2.4–13.3]) even after adjustment for LVEF <35% and atrial fibrillation. Age at diagnosis of HCM-LVSD had a modest effect (HR 1.1 [95% CI, 1.0–1.1] per 5-year increment). Sex, NYHA class III/IV at HCM-LVSD diagnosis, and prior SRT were not independently predictive of the composite outcome.

### Incident Development of HCM-LVSD

**Figure 4.**
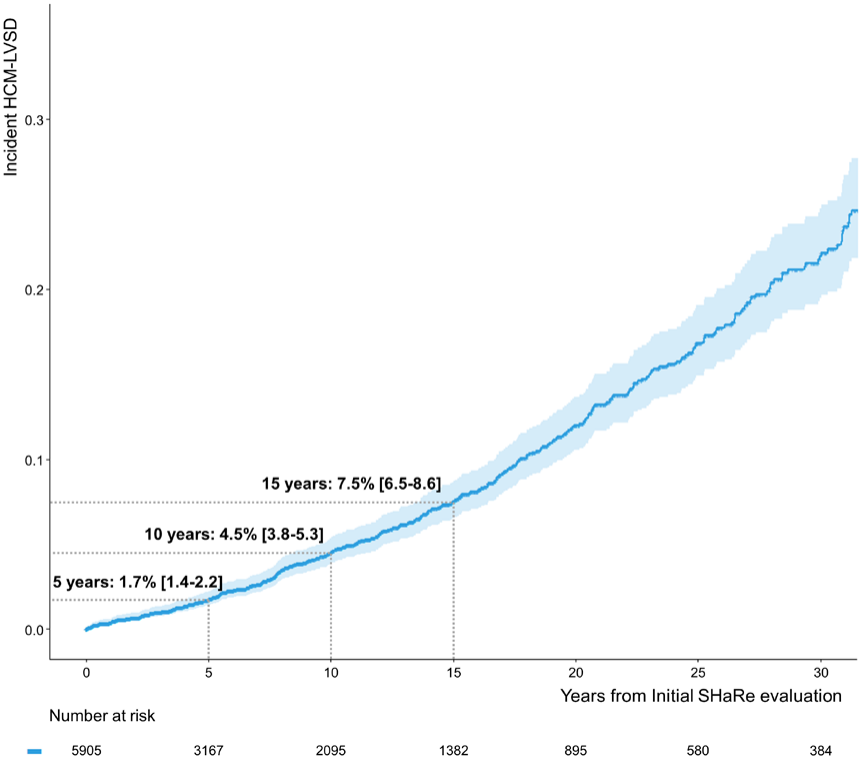
**Time from initial evaluation to developing incident hypertrophic cardiomyopathy with left ventricular systolic dysfunction (HCM-LVSD).** Rates of developing incident HCM-LVSD were 1.7% (95% CI, 1.4–2.2) at 5 years, 4.5% (95% CI, 3.8–5.3) at 10 years, and 7.5% (95% CI, 6.5–8.6) at 15 years. SHaRe indicates Sarcomeric Human Cardiomyopathy Registry.

A cohort of 5905 patients with longitudinal data and LVEF >50% at the initial evaluation was available to analyze incident development of HCM-LVSD. Of those, 350 (5.9%) developed HCM-LVSD during follow-up. The estimated incidence rates were 0.5%/y from initial evaluation, 1.7% (95% CI, 1.4%–2.2%) at 5 years, 4.5% (3.8%–5.3%) at 10 years, and 7.5% (6.5%–8.6%) at 15 years after the initial SHaRe visit (Figure 4).

**Figure 5.**
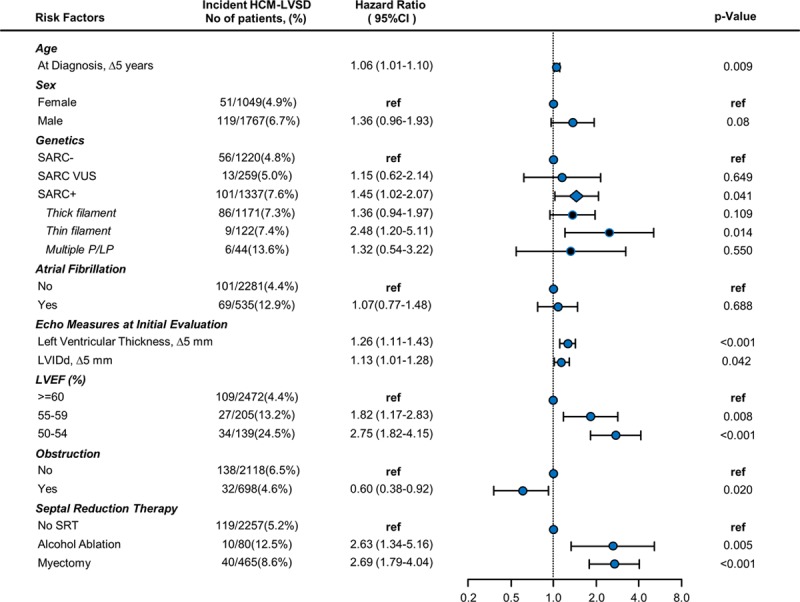
**Forest plot depicting risk predictors for developing incident hypertrophic cardiomyopathy with left ventricular systolic dysfunction (HCM-LVSD).** Unadjusted number of events per variable is shown in second column. Cox proportional hazards model is based on 2816 patients, of whom170 developed incident HCM-LVSD. Hazard ratios are adjusted for all included risk factors. The diamond symbol in the cohort with ≥1 pathogenic/likely pathogenic variant (SARC+) represents the composite effect of thin filament, thick filament, and multiple pathogenic/likely pathogenic (P/LP) sarcomeric variants. LVEF indicates left ventricular ejection fraction; LVIDd, left ventricular diastolic diameter; ref, referent; SARC−, no clinically relevant sarcomere variants identified on genetic testing; SARC VUS, a variant of unknown significance in a sarcomere gene; and SRT, septal reduction therapy.

A Cox proportional hazards model was performed to identify predictors of developing HCM-LVSD. Of the 5905 patients with available follow-up, 3524 had genetic data available and no history of SRT at initial evaluation. Complete echocardiographic data were available in 2627 of those, and in 189 patients, data were imputed with a median time of 87 days (interquartile range, 39–380 days) between the initial evaluation and echocardiography. Hence, the model was based on a cohort of 2816 patients with HCM, with 170 outcomes of incident HCM-LVSD (Figure 5). Greater LV cavity size, greater LV wall thickness, and the presence of a pathogenic/likely pathogenic sarcomeric variant were associated with increased risk of incident HCM-LVSD, with HRs ranging from 1.2 to 1.5. The presence of a variant in a thin filament gene (*TNNT2*, *TNNI3*, *TPM1*, *ACTC*) was associated with an HR of 2.5 (95% CI, 1.2–5.1). Borderline LVSD at the initial SHaRe evaluation was associated with a higher risk of incident HCM-LVSD (LVEF, 55%–59%: HR, 1.8 [95% CI, 1.2–2.8]; LVEF 50%–54%: HR, 2.8 [95% CI, 1.8–4.2]), likely identifying patients with incipient HCM-LVSD remodeling at presentation. In patients with a baseline LVEF between 50% and 59% who later developed HCM-LVSD, the median time to onset of HCM-LVSD was 3.4 years (2.32–4.23 years) if LVEF was 55% to 59% and 2.2 years (1.80–3.2 years) if LVEF was 50% to 54%.

Although atrial fibrillation was a predictor of prognosis and was more frequently seen in patients with HCM-LVSD (11.3% versus 4.3%), it was not an independent risk predictor for the development of incident HCM-LVSD remodeling (HR, 1.1 [95% CI, 0.8–1.5]). Patients with obstructive physiology (maximal LV outflow tract gradient >30 mm Hg) were less likely to develop HCM-LVSD (HR, 0.6 [95% CI, 0.4–0.9]), suggesting that obstructive physiology was not driving the development of systolic dysfunction in the majority of patients with HCM-LVSD.

A full list of multiple pathogenic/like pathogenic sarcomeric variants is provided in Table I in the Data Supplement. Although information about medication use is not fully captured in SHaRe, in an assessment of the subset of patients with documented medication use (2300 of 2816 for the model predicting incident risk and 289 of 394 for the prognosis model), neither β-blocker nor calcium channel blocker use had a significant association with risk for developing incident HCM-LVSD or prognosis with HCM-LVSD once developed.

Cardiac magnetic resonance studies were available in a subset of 2751 patients (HCM without LVSD, n=2590; incident HCM-LVSD, n=105; prevalent HCM-LVSD, n=56; Table II in the Data Supplement). Late gadolinium enhancement (LGE) was more prevalent in combined patients with HCM-LVSD compared with patients without LVSD (85.6% versus 69.5%; *P*<0.001). In a Cox regression model adjusted for genotype, age at diagnosis, cardiac magnetic resonance LVEF, and sex, the presence of LGE was associated with an HR of 2.3 (95% CI, 1.0–4.9; *P*=0.039) for developing incident HCM-LVSD.

### Impact of SRT

Given the observation of a higher prevalence of SRT in patients with HCM-LVSD, we included SRT in the model assessing incident development of HCM-LVSD (Figure 5). An increased risk for developing incident HCM-LVSD was seen in patients who previously had a myectomy (n=40 of 465; HR, 2.7 [95% CI, 1.8–4.0]) or alcohol ablation (n=10 of 80; HR, 2.6 [95% CI, 1.3–5.2]). Further analyses were then pursued to better characterize the association between SRT and incident development of systolic dysfunction. Overall, 1221 patients in SHaRe underwent SRT, of whom 138 (11.4%) developed incident HCM-LVSD. These 138 patients were a mean 43±18 years of age at SRT and developed HCM-LVSD a median of 5.6 years (3.9–8.6 years) after SRT.

A sensitivity analysis was performed that excluded patients who underwent SRT before the year 2000 (n=8) to limit analyses to patients whose procedures were performed in the current era. Results were similar to the full model (HR for SRT, 2.4 [95% CI, 1.7–3.6]), suggesting that the association between HCM-LVSD and myectomy was not driven primarily by patients who underwent procedures in the remote past. In addition, adjusting for participating site did not change the estimated risk (data not shown).

We compared baseline clinical characteristics between patients with HCM-LVSD who did and those who did not undergo SRT. Patients with HCM-LVSD with prior SRT were similar in age, sex, sarcomeric variant status, and presenting LV wall thickness and LV cavity size, but they had larger left atrial diameter and greater LVEF at baseline evaluation (Table III in the Data Supplement). Patients with HCM-LVSD with prior SRT were also more likely to have NYHA class III/IV symptoms at presentation with HCM-LVSD.

We postulated that the development of HCM-LVSD after myectomy could be the result of procedure-related left bundle-branch block, leading to a mild to moderate reduction in LVEF (LVEF, 35%–49%) but no other clinical sequelae (no atrial fibrillation, appropriate ICD therapy, or NYHA class III/IV symptoms, denoted uncomplicated HCM-LVSD). No significant difference in the proportion of uncomplicated HCM-LVSD was found between patients with and those without a history of SRT (22 of 105 [20.9%] versus 105 of 402 [26.1%]; *P*=0.31), suggesting that systolic dysfunction in patients with prior myectomy is not purely a reflection of otherwise inconsequential bundle-branch block.

Once HCM-LVSD developed, the natural history in patients with prior SRT was not significantly different from that in patients without prior SRT (Table IV in the Data Supplement). Among patients with HCM-LVSD with prior SRT, 26.8% developed LVEF <35% (versus 36.5% without prior SRT; *P*=0.051). Patients with prior SRT were more likely to have an ICD implanted but less likely to have appropriate firing. No significant differences were found for death, need for cardiac transplantation, LVAD, or atrial fibrillation between patients with HCM-LVSD with and those without prior SRT.

## Discussion

This study leverages a large, international cohort to better characterize the prevalence and natural history of systolic dysfunction in HCM. The major findings are the following: First, ≈8% of patients with HCM develop systolic dysfunction with LVEF <50% (7.5% incidence over 15 years). Second, the natural history of HCM-LVSD was variable, but ≈75% of patients experienced clinically relevant adverse events, including 35% experiencing death, cardiac transplantation, or LVAD implantation an estimated median of 8.4 years after developing systolic dysfunction. Third, atrial fibrillation, LVEF <35%, and the presence of multiple sarcomeric variants were independently associated with poor prognosis. Last, sarcomeric variants (particularly in thin filament genes), borderline low baseline LVEF (50%–60%), and LGE on cardiac magnetic resonance were independently associated with incident development of HCM-LVSD.

### Prevalence and Prognosis of HCM-LVSD

Earlier, smaller studies on HCM-LVSD (n=20–156) have reported a prevalence ranging from 4% to 9% and a malignant natural history, with ≈60% of patients with HCM-LVSD experiencing death or cardiac transplantation over a mean time of only 2.7 to 5 years from recognition of systolic dysfunction.^6–8,13–15^

In our cohort of 6793 patients with HCM with a median follow-up of >3 years at HCM specialty centers, 553 developed systolic dysfunction, representing a prevalence of 8.1%. Our results confirm that systolic dysfunction confers adverse outcomes. Seventy-five percent of patients with HCM-LVSD experienced adverse sequelae, and they were at least twice as likely as patients without systolic dysfunction to have NYHA class III/IV symptoms, appropriate ICD therapy, atrial fibrillation, and stroke. One-third of patients with HCM-LVSD developed severe LVSD (LVEF <35%), and 35% experienced a death equivalent as reflected by the composite outcome of death, cardiac transplantation, or LVAD implantation. Compared with patients with HCM without systolic dysfunction, mortality during follow-up was >2-fold higher in patients with HCM-LVSD; the need for cardiac transplantation or LVAD was >11- and 26-fold higher, respectively. However, our findings indicate that the time frame is less precipitous for most individuals with HCM-LVSD than previously described. On average, systolic dysfunction developed a median of 15 years after initial diagnosis of HCM. The median time to death, cardiac transplantation, or LVAD implantation was 8.4 years after recognition of systolic dysfunction and 11.4 years to death alone. Atrial fibrillation (HR, 2.6 [95% CI, 1.7–3.8]), LVEF <35% (HR, 2.0 [95% CI, 1.3–2.8]), and the presence of multiple pathogenic sarcomeric variants (HR, 5.6 [95% CI, 2.1–13.5]) were independent predictors of poor prognosis, as assessed by the composite outcome of death, cardiac transplantation, or LVAD implantation.

Moreover, the experience of patients with HCM-LVSD was quite broad, with many free from serious events for many years after the initial decline in LVEF was documented. Thus, end stage, the previously used nomenclature, does not accurately reflect the majority of patients with HCM-LVSD. Further investigation is needed to more fully understand this heterogeneity and to characterize the underlying factors that drive progressive adverse remodeling associated with decreased LV systolic function in HCM.

### Incident Development of Systolic Dysfunction

A multivariable regression model identified sarcomeric variants overall (HR, 1.5 [95% CI, 1.0–2.1]) and thin filament variants in particular (HR, 2.5 [95% CI, 1.2–5.1]), LVEF 50% to 60% (HR, 1.8–2.8), and greater LV wall thickness (HR, 1.3 [95% CI, 1.1–1.4] per 5-mm increment) as independent predictors for developing systolic dysfunction. The transition phase of an LVEF 50% to 59% was described by Olivotto et al^16^ in 2010. They also identified a greater extent of myocardial fibrosis associated with declining LVEF.^16^ Similarly, our study identified the presence of late gadolinium and LVEF of 50% to 60% as independent predictors for incident systolic dysfunction. Although this LVEF would be considered within normal range for other populations, it appears to identify patients with HCM with a nearly 3-fold increased risk of developing HCM-LVSD who would likely benefit from closer management.

Our data identified a potential association between invasive SRT and future development of HCM-LVSD. This signal (HR >2.6 for surgical myectomy and alcohol ablation) persisted even after considering LV morphology, function, and obstructive physiology before the procedure, atrial fibrillation, participating site, and limiting the analysis to procedures performed in the current era (since 2000). In our cohort, HCM-LVSD developed in 11% of patients who underwent SRT ≈6 years after the procedure was performed. Patients with HCM-LVSD who underwent prior SRT were more likely to have an ICD implanted than those without prior SRT, presumably related to procedure-associated conduction block, but appropriate ICD therapies did not differ significantly. Prognosis and clinical outcomes appeared similar in patients with HCM-LVSD with and without prior SRT.

Septal reduction therapies have been studied extensively for safety and efficacy of symptom relief. In particular, surgical myectomy has been shown to be associated with low morbidity and mortality in experienced centers such as SHaRe sites.^17–19^ SRT plays an important role in the clinical management of HCM, providing highly effective relief of symptoms resulting from obstructive physiology. However, prior studies on SRT were not designed to identify a potential association between myectomy and future development of HCM-LVSD. Thus, they would not have captured the development of systolic dysfunction or death-equivalent outcomes of cardiac transplantation or LVAD implantation (30 patients in our cohort). Nonetheless, 2 smaller studies also identified an increased risk of developing impaired LV function after SRT^20^ and emphasized that the duration of follow-up needs to be relatively long, at least 8 to 10 years, to capture this adverse remodeling.^21^ This time frame is supported by our current findings. Similarly, all but 1 published study focusing on HCM-LVSD systematically excluded patients who underwent SRT.^5,13,16^ The study by Harris et al^6^ included 89 patients with a myectomy, 7 (8%) of whom developed HCM-LVSD, compared with 3% of patients who did not undergo myectomy. A value of *P*=0.84 was reported, without adjustment for age, sex, time to event, or disease severity.^6^

We caution that our findings reflect a relatively small number of patients who underwent SRT before developing HCM-LVSD. Although potential causal mechanisms underlying this association cannot be inferred from this retrospective analysis, we have considered several possibilities that could be explored in future studies. First, we speculate that intraventricular conduction delay/dyssynchrony related to the procedure may play a role, in which case early resynchronization therapy might be considered in certain patients (eg, LVEF <55%). Second, the HCM-LVSD cohort with SRT may be confounded by some degree of selection bias because these patients may have been referred for a higher degree of symptoms as a result of more severe underlying adverse remodeling. Last, it is possible that preexisting pressure overload could lead to more advanced remodeling in a subset of patients, despite adequate relief of LV outflow tract obstruction. Our data suggest that patients undergoing SRT require longitudinal monitoring of cardiac function after the procedure, particularly younger patients with sarcomere gene mutations. Further study is needed to better characterize the associations between SRT and HCM-LVSD, to elucidate broader effects of SRT on long-term cardiac remodeling, and to fully understand the clinical implications.

### Limitations

Although SHaRe incorporates curated, longitudinal data, it is subject to the limitations inherent to all observational and partially retrospective registry-based studies. Cardiac magnetic resonance data are derived from site-based clinical reports, which do not routinely capture data on quantification and location of LGE. On the basis of recent literature, we anticipate that <4% of patients with HCM will have extensive LGE (>15% involvement).^22^ Because participating sites are high-volume HCM specialty centers, our study could be influenced by referral bias, resulting in an overestimation of the prevalence of HCM-LVSD and adverse prognosis of HCM-LVSD. Conversely, the registry is also subject to survival bias because patients must survive until being seen at a SHaRe site. It is notable that the patients with incident systolic dysfunction had longer follow-up compared with the group who did not develop systolic dysfunction, suggesting that the prevalence is likely to be higher with longer follow-up. Continued multicenter study in adequately powered cohorts is needed for more definitive understanding of HCM-LVSD.

### Conclusions

LVSD develops in ≈8% of patients with HCM and carries important clinical implications in that ≈75% of patients with HCM-LVSD will experience atrial fibrillation, stroke, advanced heart failure (LVEF <35% or NYHA class III/IV), appropriate ICD therapy, cardiac transplantation, LVAD implantation, or death. However, the natural history appears to evolve over a number of years, and individual patient experience is variable. Genetic substrate appears to play a role in both the risk for developing systolic dysfunction and prognosis after systolic dysfunction is present. In addition, patients with HCM with an LVEF between 50% and 60% had a nearly 3-fold increased risk of developing systolic dysfunction and may benefit from closer clinical surveillance. Further study is required to refine clinical predictors of progressive adverse remodeling, especially in patients with prior SRT, and to more fully characterize the determinants of clinical outcomes.

## Acknowledgments

The authors are grateful for the dedicated work of site data managers Kermshlise Picard, Hoshang Farhad, Efhalia Kaynor, and Kavitha Nutakki (Brigham and Women’s Hospital); Pieter Vriesendorp and Hannah van Velzen (Erasmus Medical Center); Fausto Barlocco (Careggi University Hospital); Alexandra Butters (Centenary Institute); and Maryann Concannon (University of Michigan).

## Sources of Funding

Funding for SHaRe has been provided through an unrestricted research grant from MyoKardia, Inc, a startup company that is developing therapeutics that target the sarcomere. MyoKardia, Inc had no role in approving the content of this article. Dr Ho is supported by funding from the National Institutes of Health (1P50HL112349 and 1U01HL117006). Dr Marstrand is supported by funding from The Danish Heart Foundation (grant 17-R115-A7532-22065), Elite Research Travel Grant from the Danish Ministry of Higher Education and Science, The FUKAP Foundation, Torben og Alice Frimodts Fond, Augustinus fonden, and Knud Højgaards fond. Dr Day is supported by funding from the National Institutes of Health (R01 GRANT11572784). Dr Olivotto is supported by the Italian Ministry of Health (Left Ventricular Hypertrophy in Aortic Valve Disease and Hypertrophic Cardiomyopathy: Genetic Basis, Biophysical Correlates and Viral Therapy Models [RF-2013-02356787] and NET-2011-02347173 [Mechanisms and Treatment of Coronary Microvascular Dysfunction in Patients With Genetic or Secondary Left Ventricular Hypertrophy]) and by the Tuscany Registry of Sudden Cardiac Death project (FAS-Salute 2014, Regione Toscana). Dr Ware is supported by the Wellcome Trust (107469/Z/15/Z) and the Medical Research Council (United Kingdom). Dr Semsarian is the recipient of a National Health and Medical Research Council Practitioner Fellowship (No. 1154992).

## Disclosures

Drs Ho, Day, Olivotto, Colan, Ashley, and Ware receive research support and/or consulting fees from MyoKardia, Inc. The other authors report no conflicts.

## Supplementary Material



## References

[1] Elliott PM, Anastasakis A, Borger MA, Borggrefe M, Cecchi F, Charron P, Hagege AA, Lafont A, Limongelli G, Mahrholdt H (2014). 2014 ESC guidelines on diagnosis and management of hypertrophic cardiomyopathy: the Task Force for the Diagnosis and Management of Hypertrophic Cardiomyopathy of the European Society of Cardiology (ESC).. Eur Heart J.

[2] Gersh BJ, Maron BJ, Bonow RO, Dearani JA, Fifer MA, Link MS, Naidu SS, Nishimura RA, Ommen SR, Rakowski H, American College of Cardiology Foundation/American Heart Association Task Force on Practice Guidelines; American Association for Thoracic Surgery; American Society of Echocardiography; American Society of Nuclear Cardiology; Heart Failure Society of America; Heart Rhythm Society; Society for Cardiovascular Angiography and Interventions; Society of Thoracic Surgeons (2011). 2011 ACCF/AHA guideline for the diagnosis and treatment of hypertrophic cardiomyopathy: executive summary: a report of the American College of Cardiology Foundation/American Heart Association Task Force on Practice Guidelines.. Circulation.

[3] Semsarian C, Ingles J, Maron MS, Maron BJ (2015). New perspectives on the prevalence of hypertrophic cardiomyopathy.. J Am Coll Cardiol.

[4] Alfares AA, Kelly MA, McDermott G, Funke BH, Lebo MS, Baxter SB, Shen J, McLaughlin HM, Clark EH, Babb LJ (2015). Results of clinical genetic testing of 2,912 probands with hypertrophic cardiomyopathy: expanded panels offer limited additional sensitivity.. Genet Med.

[5] Thaman R, Gimeno JR, Murphy RT, Kubo T, Sachdev B, Mogensen J, Elliott PM, McKenna WJ (2005). Prevalence and clinical significance of systolic impairment in hypertrophic cardiomyopathy.. Heart.

[6] Harris KM, Spirito P, Maron MS, Zenovich AG, Formisano F, Lesser JR, Mackey-Bojack S, Manning WJ, Udelson JE, Maron BJ (2006). Prevalence, clinical profile, and significance of left ventricular remodeling in the end-stage phase of hypertrophic cardiomyopathy.. Circulation.

[7] Spirito P, Maron BJ, Bonow RO, Epstein SE (1987). Occurrence and significance of progressive left ventricular wall thinning and relative cavity dilatation in hypertrophic cardiomyopathy.. Am J Cardiol.

[8] Fernández A, Vigliano CA, Casabé JH, Diez M, Favaloro LE, Guevara E, Favaloro RR, Laguens RP (2011). Comparison of prevalence, clinical course, and pathological findings of left ventricular systolic impairment versus normal systolic function in patients with hypertrophic cardiomyopathy.. Am J Cardiol.

[9] Galati G, Leone O, Pasquale F, Olivotto I, Biagini E, Grigioni F, Pilato E, Lorenzini M, Corti B, Foà A (2016). Histological and histometric characterization of myocardial fibrosis in end-stage hypertrophic cardiomyopathy.. Circ Hear Fail.

[10] Ho CY, Day SM, Ashley EA, Michels M, Pereira AC, Jacoby D, Cirino AL, Fox JC, Lakdawala NK, Ware JS (2018). Genotype and lifetime burden of disease in hypertrophic cardiomyopathy: insights from the Sarcomeric Human Cardiomyopathy Registry (SHaRe).. Circulation.

[11] Richards S, Aziz N, Bale S, Bick D, Das S, Gastier-Foster J, Grody WW, Hegde M, Lyon E, Spector E, ACMG Laboratory Quality Assurance Committee (2015). Standards and guidelines for the interpretation of sequence variants: a joint consensus recommendation of the American College of Medical Genetics and Genomics and the Association for Molecular Pathology.. Genet Med.

[12] Peduzzi P, Concato J, Feinstein AR, Holford TR (1995). Importance of events per independent variable in proportional hazards regression analysis, II: accuracy and precision of regression estimates.. J Clin Epidemiol.

[13] Biagini E, Coccolo F, Ferlito M, Perugini E, Rocchi G, Bacchi-Reggiani L, Lofiego C, Boriani G, Prandstraller D, Picchio FM (2005). Dilated-hypokinetic evolution of hypertrophic cardiomyopathy: prevalence, incidence, risk factors, and prognostic implications in pediatric and adult patients.. J Am Coll Cardiol.

[14] Pasqualucci D, Fornaro A, Castelli G, Rossi A, Arretini A, Chiriatti C, Targetti M, Girolami F, Corda M, Orrù P (2015). Clinical spectrum, therapeutic options, and outcome of advanced heart failure in hypertrophic cardiomyopathy.. Circ Heart Fail.

[15] Biagini E, Olivotto I, Iascone M, Parodi MI, Girolami F, Frisso G, Autore C, Limongelli G, Cecconi M, Maron BJ (2014). Significance of sarcomere gene mutations analysis in the end-stage phase of hypertrophic cardiomyopathy.. Am J Cardiol.

[16] Olivotto I, Maron BJ, Appelbaum E, Harrigan CJ, Salton C, Gibson CM, Udelson JE, O’Donnell C, Lesser JR, Manning WJ (2010). Spectrum and clinical significance of systolic function and myocardial fibrosis assessed by cardiovascular magnetic resonance in hypertrophic cardiomyopathy.. Am J Cardiol.

[17] Nguyen A, Schaff HV, Nishimura RA, Geske JB, Ackerman MJ, Bos JM, Dearani JA, Ommen SR (2019). Survival after myectomy for obstructive hypertrophic cardiomyopathy: what causes late mortality?. Ann Thorac Surg.

[18] Meghji Z, Nguyen A, Fatima B, Geske JB, Nishimura RA, Ommen SR, Lahr BD, Dearani JA, Schaff HV (2019). Survival differences in women and men after septal myectomy for obstructive hypertrophic cardiomyopathy.. JAMA Cardiol.

[19] Woo A, Williams WG, Choi R, Wigle ED, Rozenblyum E, Fedwick K, Siu S, Ralph-Edwards A, Rakowski H (2005). Clinical and echocardiographic determinants of long-term survival after surgical myectomy in obstructive hypertrophic cardiomyopathy.. Circulation.

[20] Parbhudayal RY, Güçlü A, Zweerink A, Biesbroek PS, Croisille P, Clarysse P, Michels M, Stooker W, Vonk ABA, van der Ven PM (2019). Myocardial adaptation after surgical therapy differs for aortic valve stenosis and hypertrophic obstructive cardiomyopathy.. Int J Cardiovasc Imaging.

[21] Turina J, Jenni R, Krayenbuehl HP, Turina M, Rothlin M (1986). Echocardiographic findings late after myectomy in hypertrophic obstructive cardiomyopathy.. Eur Heart J.

[22] Neubauer S, Kolm P, Ho CY, Kwong RY, Desai MY, Dolman SF, Appelbaum E, Desvigne-Nickens P, DiMarco JP, Friedrich MG, HCMR Investigators (2019). Distinct subgroups in hypertrophic cardiomyopathy in the NHLBI HCM Registry.. J Am Coll Cardiol.

