# Supplementary material for: Hypertrophic Cardiomyopathy With Left Ventricular Systolic Dysfunction: Insights From the SHaRe Registry
Source: Circulation. 2020 Mar 31;141(17):1371–83. doi: 10.1161/CIRCULATIONAHA.119.044366 (PMC7182243; doi:10.1161/CIRCULATIONAHA.119.044366)
Supplement: Supplementary file 1 [file cir-141-1371-s001.pdf]

SUPPLEMENTAL MATERIAL

Supplemental Table 1 – List of Sarcomeric Variants in Patients with HCM and Multiple Pathogenic/Likely Pathogenic Variants. Of the 63 patients, 13 (21%) had HCM-LVSD.
P= Pathogenic, LP= Likely Pathogenic, VUS= Variant of Unknown Significance.

Patient	HCM-LVSD at anytime	Sarcomeric Variants
1	Yes	MYBPC3:P p.Glu258Lys(c.772G>A); MYH7:P p.Arg723Cys(c.2167C>T)
2	Yes	MYBPC3:P p.Glu258Lys(c.772G>A); MYH7:P p.Arg723Cys(c.2167C>T)
3	Yes	MYBPC3:LP p.Arg502Trp(c.1504C>T); MYBPC3:P c.927-2A>G
4	Yes	MYBPC3:LP p.Gly531Arg(c.1591G>C); TNNT2:LP p.Phe110Leu(c.330T>G)
5	Yes	MYBPC3:LP p.Pro371Arg(c.1112C>G); MYBPC3:P p.Lys1065Glufs*12(c.3192dup); MYH7:P p.Arg869His(c.2606G>A)
6	Yes	MYBPC3:P p.Glu258Lys(c.772G>A); MYH7:P p.Arg869His(c.2606G>A); TNNI3:P p.Ala86Glyfs*23(c.257_258del)
7	Yes	MYBPC3:LP p.Pro371Arg(c.1112C>G); MYBPC3:P p.Lys1065Glufs*12(c.3192dup)
8	Yes	MYH7:P p.Gly768Arg(c.2302G>A); TNNT2:P p.Arg285Cys(c.853C>T)
9	Yes	MYBPC3:LP p.Pro371Arg(c.1112C>G); MYBPC3:VUS p.Arg970Trp(c.2908C>T); MYBPC3:P p.Lys1065Glufs*12(c.3192dup)
10	Yes	MYBPC3:LP p.Pro371Arg(c.1112C>G); MYBPC3:P p.Lys1065Glufs*12(c.3192dup)
11	Yes	MYBPC3:P c.3628-41_3628-17del;TNNI3:LP p.Arg162Trp(c.484C>T);TNNI3:LP p.Arg162Trp(c.484C>T)
12	Yes	MYBPC3:P p.Tyr797*(c.2391C>A);MYBPC3:P p.Tyr797*(c.2391C>A);PLN:VUS c.2T>C
13	Yes	DSP:VUS c.3956C>G;MYBPC3:PAT p.Tyr797*(c.2391C>A);MYBPC3:P p.Tyr797*(c.2391C>A);PLN:VUS c.2T>C;TTN:VUS c.67330C>T;TTN:VUS c.89714G>A
14	No	MYBPC3:LP p.Pro371Arg(c.1112C>G);MYBPC3:P p.Lys1065Glufs*12(c.3192dup)
15	No	ACTN2:VUS p.Arg572Trp(c.1714C>T);MYBPC3:P p.Arg502Gln(c.1505G>A);MYBPC3:P c.3190+1G>A;TNNT2:P p.Lys210del(c.629_631del)
16	No	LAMA4:p.Ala276Asp(c.827_828delinsAC);LAMA4:P p.Ala276Asp(c.827_828delinsAC); TNNT2:PAT p.Trp287*(c.860G>A);TNNT2:PAT p.Trp287*(c.860G>A)
17	No	MYBPC3:P p.Trp818*(c.2524:G>A);MYL3:P p.Arg154His(c.461:G>A)
18	No	MYBPC3:P p.Pro955Argfs*95(c.2864_2865del);MYH7:P p.Gly1057Asp(c.3170G>A)
19	No	MYH7:P p.Ala797Thr(c.2389G>A);MYH7:LP p.Arg807His(c.2420G>A)
20	No	MYH7:LP p.Glu894Gly(c.2681A>G);MYH7:P p.Glu924Lys(c.2770G>A)
21	No	MYBPC3:P p.Gln1233*(c.3697C>T);MYBPC3:P p.Gln1233*(c.3697C>T)

22	No	MYBPC3:P c.2308+1G>A;MYH7:P p.Val606Met(c.1816G>A)
23	No	MYBPC3:P p.Pro955Argfs*95(c.2864del);MYBPC3:P c.927-9G>A
24	No	MYH7:P c.3100-2A>C;MYH7:LP p.Arg1712Gln(c.5135G>A)
25	No	MYBPC3:P p.Glu334*(c.1000G>T);MYL2:P p.Glu22Lys(c.64G>A)
26	No	MYBPC3:P p.Glu258Lys(c.772G>A);MYH7:P p.Arg694Cys(c.2080C>T)
27	No	MYBPC3:P c.772G>C;MYH7:LP p.Lys865Arg(c.2594A>G)
28	No	MYBPC3:P p.Ala392Leufs*14(c.1174del); MYBPC3:LP p.Arg597Trp(c.1789C>T)
29	No	MYBPC3:P p.Glu542Gln(c.1624G>C);TNNT2:LP p.Lys66Gln(c.196A>C)
30	No	MYBPC3:LP p.Pro371Arg(c.1112C>G);MYBPC3:P p.Lys1065Glufs*12(c.3192dup)
31	No	MYBPC3:LP p.Pro371Arg(c.1112C>G);MYBPC3:P p.Lys1065Glufs*12(c.3192dup)
32	No	MYH7:P p.Thr177Ile(c.530C>T);MYH7:LP p.Ile303Met(c.909C>G)
33	No	MYBPC3:P p.Ala392Leufs*14(c.1174del);MYL3:LP p.Gly146Trpfs*7(c.435dup)
34	No	MYBPC3:P p.Glu258Lys(c.772G>A);TNNI3:P p.Ala86Glyfs*23(c.257_258del)
35	No	MYBPC3:LP p.Pro371Arg(c.1112C>G);MYBPC3:P p.Lys1065Glufs*12(c.3192dup)
36	No	MYBPC3:P p.Ala392Leufs*14(c.1174del);MYBPC3:LP p.Arg597Trp(c.1789C>T)
37	No	MYBPC3:P p.Glu258Lys(c.772G>A);MYH7:LP p.Lys865Arg(c.2594A>G)
38	No	MYH7:LP p.Met690Thr(c.2069T>C);MYH7:LP p.Asp1869Gly(c.5606A>G)
39	No	MYH7:P p.Arg869His(c.2606G>A);TNNT2:P p.Arg286Cys(c.856C>T)
40	No	MYBPC3:P c.506-2A>C;MYH7:P p.Arg723Cys(c.2167C>T)
41	No	MYBPC3:P c.506-2A>C;MYH7:P p.Arg723Cys(c.2167C>T)
42	No	MYH7:P p.Gly768Arg(c.2302G>A);TNNT2:P p.Arg285Cys(c.853C>T)
43	No	MYBPC3:P p.Glu258Lys(c.772G>A);TNNT2:P p.Arg285Cys(c.853C>T)
44	No	MYBPC3:P p.Glu258Lys(c.772G>A);MYH7:P p.Arg869His(c.2606G>A)
45	No	MYBPC3:P p.Glu258Lys(c.772G>A);TNNT2:P p.Arg285Cys(c.853C>T)
46	No	MYBPC3:LP p.Pro371Arg(c.1112C>G);MYBPC3:P p.Lys1065Glufs*12(c.3192dup)
47	No	MYBPC3:LP p.Pro371Arg(c.1112C>G);MYBPC3:P p.Lys1065Glufs*12(c.3192dup)
48	No	MYBPC3:P p.Ala392Leufs*14(c.1174del);MYBPC3:LP p.Arg597Trp(c.1789C>T)
49	No	MYBPC3:P p.Glu258Lys(c.772G>A);TNNT2:P p.Arg285Cys(c.853C>T)
50	No	MYH7:P p.Ala797Thr(c.2389G>A);MYH7:P p.Gly853fs(c.2558del)
51	No	MYBPC3:P p.Pro955Argfs*95(c.2864_2865del); TNNI3:P p.Ala157Val(c.470C>T)
52	No	MYBPC3:LP p.Arg502Trp(c.1504C>T); MYBPC3:P c.927-9G>A
53	No	MYH7:P p.Ala226Val(c.677C>T);MYH7:P p.Ala226Val(c.677C>T)

54	No	MYH7:LP p.His581Arg(c.1742A>G); MYH7:P p.Gly584Ser(c.1750G>A)
55	No	MYH7:LP p.His581Arg(c.1742A>G); MYH7:P p.Gly584Ser(c.1750G>A)
56	No	MYBPC3:P p.Trp792Valfs*41(c.2373insG); MYH7:P p.Arg694Cys(c.2080C>T)
57	No	MYBPC3:P p.Trp792Valfs*41(c.2373insG); MYH7:P p.Arg694Cys(c.2080C>T)
58	No	MYBPC3:LP p.Arg810Leu(c.2429G>T); TNNI3:P p.Arg162Gln(c.485G>A)
59	No	MYBPC3:P p.Phe305Profs*27(c.913_914del); MYH7:LP p.Asn1664Lys(c.4992C>A)
60	No	MYBPC3: Pp.Leu1221Argfs*16(c.3662_3662del); MYH7:LP p.His1524Arg(c.4571A>G)
61	No	MYBPC3: Pc.927-9G>A; MYBPC3:P c.927-9G>A
62	No	MYBPC3:LPp.Val388Met(c.1012G>A); MYH7:LP p.Val338Met(c.1012G>A)
63	No	MYBPC3:P p.Glu60fs*49(c.177_187del); MYBPC3:P p.Glu1096*(c.3288G>A)
64	No	MYH7:LP p.Arg143Gln(c.428G>A); TNNT2:P p.Trp287*(c.860G>A)
65	No	MYL3:P p.Ala57Gly(c.170C>G); TNNI3:LP p.Arg145Gln(c.434G>A)
66	No	MYBPC3:LP p.Arg810His(c.2429G>A); MYH7:LP p.Arg1712Gln(c.5135G>A)
67	No	MYBPC3:VUS p.His947Asn(c.2839C>A);MYBPC3:P p.Ala181Cysfs*53(c.540_559del);MYH7:LP p.Arg204His(c.611G>A)
68	No	MYBPC3:LPp.Pro371Arg(c.1112C>G);MYBPC3:Pp.Lys1065Glufs*12(c.3192dup)

Supplemental Table 2 – Clinical Characteristics at Time of First MRI. Patients were considered Prevalent HCM-LVSD if they had a history of LVEF below 50 on an echocardiography.

**Demographics at first MRI**	**Total N = 2,751**	**HCM without LVSD N = 2,590**	**P value** HCM without LVSD vs. Incident HCM-LVSD	**Incident HCM-LVSD N = 105**	**Prevalent HCM-LVSD N = 56**	**P value** Incident HCM-LVSD vs. Prevalent HCM-LVSD
Age at first MRI, mean±SD	46.20±17.90	46.2±18.0	0.955	46.1±16.4	45.1±18.6	0.726
Age at Diagnosis with HCM, years, mean±SD	41.89±18.69	42.1±20.4	0.031	35.1±19.0	32.6±18.5	0.069
Sex, male, n(%)	1,786 (64.9)	1680 (64.9)	0.941	69 (65.7)	37 (66.1)	1.000
Follow-up, years, median [IQR]	3.2 [1.1-6.1]	3.1 [0.5-7.1]	<0.001	6.0 [2.8-9.9]	4.14 [2.2-6.5]	0.007
**Genetics, n(%)**			0.254*			0.802*
SARC-*	849 (40.8)	799 (45.2)		30 (37.5)	15 (31.9)	
SARC VUS*	377 (18.1)	185 (10.5)		7 (8.8)	4 (8.5)	
SARC+*	855 (41.1)	784 (44.3)		43 (53.8)	28 (59.6)	
No genetic testing	*670 (24.3)*	*822 (31.7)*		*25 (23.8)*	*9 (16.1)*	
**MRI data**						
LVEF%, mean±SD	66±10	67±10	<0.001	57±14	51±7	0.018
LVWT mm, mean±SD	21±265	21±273	0.654	12±6	13±7	0.711
LVEDV, ml, mean±SD	148±45	148±44	0.016	160±50	144±57	0.105
LGE, n(%) – yes	1695 (70.4)	1,579 (69.5)	0.002	74 (87.1)	45 (83.3)	0.717

*Percentages and P-values represent patients with genetic testing (n=2081)

Supplemental Table 3 – Clinical Characteristics at Time of Presentation with HCM-LVSD in Patients with and Without Prior Septal Reduction Therapy (excluding 13 patients who underwent SRT after developing HCM-LVSD)

**Demographics at HCM-LVSD presentation**	**Total**<br>N = 540	**HCM-LVSD Patients without prior SRT**<br>**N = 402**	**HCM-LVSD Patients with prior SRT**<br>**n = 138**	**P-value**
Sex male	341 (63.1)	257 (63.9)	84 (61.0)	0.589
Age at developing HCM-LVSD, years, mean (SD)	50.5±17.9	50.4±18.3	51.0±16.8	0.708
**Genetics, n(%)**				0.191
SARC-*	120 (31.3)	80 (29.0)	40 (37.0)	
SARC VUS*	28 (7.3)	23 (8.3)	5 (4.6)	
SARC+*	236 (61.5)	173 (62.7)	63 (58.3)	
No genetic testing	*156 (28.8)*	*126 (31.3)*	*30 (21.7)*	*0.032*
NYHA III/IV at presentation with HCM-LVSD, n (%)	155 (30.7)	100 (26.3)	55 (44.0)	<0.001
**Echo measures at HCM-LVSD presentation**				
Maximal LVWT, mm	17±6	17±5	17±6	0.749
LVEF%, mean±SD	40±8	39±8	41±7	0.032
LVEF<35%, n (%)	139 (25.7)	118 (29.4)	21 (15.2)	0.044
Obstruction, LVOT > 30 mmHg, n (%)	34 (9.5%)	21 (8.0)	13 (13.8)	0.248
LVIDd, mm, mean±SD	50±9	50±9	49±9	0.701
Left Atrial diameter, mm, mean±SD	49±1	48±1	52±1	0.005

*Percentages and P-values represent patients with genetic testing

Supplemental Table 4 – Comparison of Events in HCM-LVSD patients With and Without SRT before Developing HCM-LVSD and at Any Time (excluding 13 patients who underwent SRT after developing HCM-LVSD)

**Events**	**Total** N = 540	**HCM-LVSD without prior SRT N = 402**	**HCM-LVSD with prior SRT n = 138**	**P-value**
**Events before developing HCM-LVSD, n (%)**				
ICD implanted	213 (39.4)	147 (36.6)	66 (47.8)	0.025
Appropriate ICD firing	40 (18.8)	32 (21.7)	8 (12.1)	0.095
Atrial fibrillation	219 (41.8)	161 (41.7)	58 (42.0)	0.696
Stroke	19 (5.9)	15 (6.2)	4 (4.8)	0.818
**Events at Any Time, n (%)**				
ICD implanted	293 (54.3)	207 (51.5)	86 (62.3)	0.035
Appropriate ICD firing	73 (24.9)	59 (28.5)	14 (16.3)	0.029
Atrial fibrillation	258 (49.2)	187 (48.4)	71 (51.4)	0.612
Stroke	27 (8.3)	22 (9.2)	5 (6.0)	0.491
LVEF<35%	181 (34.0)	144 (36.5)	37 (26.8)	0.051
Death	133 (24.6)	99 (24.6)	34 (24.6)	1.000
Transplant	64 (11.9)	48 (11.9)	16 (11.6)	1.000
LVAD	10 (2.8)	8 (2.9)	2 (2.2)	1.000
